# Potential for Assessing Dynamic Problem-Solving at the Beginning of Higher Education Studies

**DOI:** 10.3389/fpsyg.2017.02022

**Published:** 2017-11-20

**Authors:** Benő Csapó, Gyöngyvér Molnár

**Affiliations:** ^1^MTA-SZTE Research Group on the Development of Competencies, University of Szeged, Szeged, Hungary; ^2^Department of Learning and Instruction, University of Szeged, Szeged, Hungary

**Keywords:** dynamic problem-solving, technology-based assessment, predictive validity, university admissions, learning strategies

## Abstract

There is a growing demand for assessment instruments which can be used in higher education, which cover a broader area of competencies than the traditional tests for disciplinary knowledge and domain-specific skills, and which measure students' most important general cognitive capabilities. Around the age of the transition from secondary to tertiary education, such assessments may serve several functions, including selecting the best-prepared candidates for certain fields of study. Dynamic problem-solving (DPS) is a good candidate for such a role, as tasks that assess it involve knowledge acquisition and knowledge utilization as well. The purpose of this study is to validate an online DPS test and to explore its potential for assessing students' DPS skills at the beginning of their higher education studies. Participants in the study were first-year students at a major Hungarian university (*n* = 1468). They took five tests that measured knowledge from their previous studies: Hungarian language and literature, mathematics, history, science and English as a Foreign Language (EFL). A further, sixth test based on the MicroDYN approach, assessed students' DPS skills. A brief questionnaire explored learning strategies and collected data on students' background. The testing took place at the beginning of the first semester in three 2-h sessions. Problem-solving showed relatively strong correlations with mathematics (*r* = 0.492) and science (*r* = 0.401), and moderate correlations with EFL (*r* = 0.227), history (*r* = 0.192), and Hungarian (*r* = 0.125). Weak but still significant correlations were found with certain learning strategies, positive correlations with elaboration strategies, and a negative correlation with memorization strategies. Significant differences were observed between male and female students; men performed significantly better in DPS than women. Results indicated the dominant role of the first phase of solving dynamic problems, as knowledge acquisition correlated more strongly with any other variable than knowledge utilization.

## Introduction

The social and economic developments of the past decades have re-launched the debate on the mission of schooling, more specifically, on the types of skills schools are expected to develop in their students in order to prepare them for an unknown future. One of the most characteristic features of these debates is a search for a new conception of the knowledge and skills students are expected to master (see e.g., Adey et al., 2007; Binkley et al., 2012; Greiff et al., 2014). These developments and expectations have reached higher education as well, and novel assessment needs have emerged to reflect the changes. Tests in higher education have traditionally been used as a part of the selection processes (entrance examinations) and to assess students' level of mastery, mostly in the form of summative tests based on the disciplinary content of courses. Recently, the functions of assessments have significantly expanded, thus requiring a renewal of assessment processes in a number of dimensions.

This study lies at the intersection of three rapidly developing fields of research on higher education. The context of the research is set by the practical needs of (1) developing new assessment methods for higher education, including innovative and efficient selection processes for choosing students for higher education studies, and assessing university outcomes beyond disciplinary knowledge and domain-specific skills. These demands have directed the attention of researchers to (2) the twenty-first-century skills as desired outcomes of higher education. Rapidly developing technology-based assessment has made it possible to measure several twenty-first-century skills and to include them in large-scale assessments. (3) Dynamic problem-solving (DPS) is one of those skills which by now has an established research background and may satisfy the needs of higher education. Solving problems in the process of being assessed in DPS based on computer-simulated scenarios involves the component skills of scientific reasoning, knowledge acquisition and knowledge utilization, all necessary for successful higher education studies (see e.g., Buchner and Funke, 1993; Funke, 2001; Greiff et al., 2012; Csapó and Funke, 2017a; Funke and Greiff, 2017). The processes of solving problems in computer-simulated scenarios involve the component skills of scientific reasoning, knowledge acquisition and knowledge utilization, all necessary for learning effectively in higher education (see e.g., Buchner and Funke, 1993; Funke, 2001; Greiff et al., 2012; Csapó and Funke, 2017a; Funke and Greiff, 2017).

As the main construct explored in the present study, DPS has already been defined and assessed in several previous studies. The problems are built on formal models represented by a finite-state automaton, where the output signals are determined by the input signal (Buchner and Funke, 1993). “In contrast to static problems, computer-simulated scenarios provide the unique opportunity to study human problem-solving and decision-making behavior when the task environment changes concurrently to subjects' actions. Subjects can manipulate a specific scenario via a number of input variables […] and they observe the system's state changes in a number of output variables. In exploring and/or controlling a system, subjects have to continuously acquire and use knowledge about the internal structure of the system” (Blech and Funke, 2005, p. 3).

In the present study, DPS was assessed with a computerized solution based on the MicroDYN approach (Greiff and Funke, 2009; Funke and Greiff, 2017) similar to that employed in delivering most interactive items in the innovative domain in PISA 2012. That assessment framework defined problem-solving in a more general way: “Problem-solving competency is an individual's capacity to engage in cognitive processing to understand and resolve problem situations where a method of solution is not immediately obvious. It includes the willingness to engage with such situations in order to achieve one's potential as a constructive and reflective citizen.” (OECD, 2013a, p. 122). An interpretation of this definition follows: “What distinguishes the 2012 assessment of problem-solving from the 2003 assessment is not so much the definition of problem-solving competency, but the mode of delivery of the 2012 assessment (computer-based) and the inclusion of problems that cannot be solved without the solver interacting with the problem situation” (OECD, 2013a, p. 122). The PISA 2012 problem-solving assessment included both static and interactive tasks, and in this context interactivity is defined as “Interactive: not all information is disclosed; some information has to be uncovered by exploring the problem situation” (OECD, 2014, p. 31, Fig. V.1.2). In the present study, all items are interactive, so the construct we assess is identical with the one PISA assessed in 2012 with its interactive items.

## Theoretical framework

### Context of the study: need for new assessments in higher education

The need to develop new assessment instruments for higher education has emerged both at international and national levels in a number of countries. There is a general intention to adapt the content of the assessments to changed expectations of the outcomes of higher education. The altered content may then require new assessment methods (see e.g., Bryan and Clegg, 2006). There is a change in the purpose of assessments as well as a visible intention to introduce the principles of evidence-based decision-making and accountability processes to higher education (Hutchings et al., 2015; Ikenberry and Kuh, 2015; Zlatkin-Troitschanskaia et al., 2015). The new functions of assessment go beyond the usual applications of summative tests to measure the mastery level of courses and include estimating educational added value of particular phases of studies, or entire training programs. As there is a great variety of competencies that are outcomes of higher education, thus limiting inter-institutional comparisons in terms of domain-specific competencies, we see a growing need to measure and compare domain-general competencies.

These intentions are clearly marked by feasibility studies launched by the OECD to compare the achievement of college and university students in a number of countries (Assessment of Higher Education Learning Outcomes, AHELO). The AHELO program included assessment of domain-specific competencies as well as of generic cognitive skills, for which the test tasks were adapted from the Collegiate Learning Assessment instrument (Tremblay et al., 2012). Another international initiative, the TUNING CALOHEE project (Measuring and Comparing Achievements of Learning Outcomes in Higher Education in Europe), intends to create an assessment system to compare the outcomes of universities in Europe (Coates, 2016).

In the United States, as the century-long history of successfully administering the Scholastic Aptitude Test (SAT) indicates, admissions processes have always been based on assessing generic cognitive skills (Atkinson and Geiser, 2009). As studies show, the SAT tests predict achievement in higher education beyond the high school grade point average. They comprise mathematical and verbal components (factor analysis with a recent version of it confirmed the two-factor model, see Wiley et al., 2014), while university admissions in many countries have usually been based on assessing domain-specific competencies (Zlatkin-Troitschanskaia et al., 2015).

A closer context of the study is Hungarian higher education and the admissions process used by its institutions. As there is no specific entrance examination, admissions are based on matriculation examination results. The matriculation examination, like so many other European countries, was introduced in Hungary in the mid-nineteenth century, and it has changed relatively little during its long history. At present, there are three mandatory subjects: (1) Hungarian language and literature, (2) mathematics, and (3) history. Beyond these, students must choose a further subject out of a large number of electives. An examination can be taken at two levels in any subject; there is an intermediate and an advanced exam. There is no exact (measurable) definition for the differences between the two levels. Intermediate exams are taken at students' schools before committees formed from teachers in their own schools, while the advanced exams are centralized and are taken before (independent) committees formed from teachers in other schools. The admissions scores are computed by complex formulas; for advanced exams, extra scores are awarded, and other factors may also be taken into account.

The inadequacy of such a selection criterion is widely discussed, but few research results are available to make evidence-based judgments about the validity of the current practice and about potential alternative solutions. It seems possible that a reformed matriculation examination could serve to certify completion of secondary studies and at the same time could act as a major component of the admissions process (Csapó, 2009). Such a matriculation examination should measure students' knowledge at one level but on a scale which represents a broad range of achievement, should be a technology-based assessment (possibly using item banks and adaptive testing), and should include a few (probably five) compulsory subjects without electives.

The new admissions processes are expected to provide a better prediction of students' success in a changed world of higher education than those of the traditional methods introduced so many decades ago. Assessment of generic cognitive skills, possibly a representative member of the twenty-first-century skills, could also be a component of a new admissions process. To explore the feasibility and validity of such an admissions model, we have measured five domain-specific competencies plus dynamic problem-solving, and we report the results in the present study.

### Definition and technology-based assessment of twenty-first-century skills in educational settings

A number of studies have analyzed the requirements of the knowledge-based economy and concluded that science, technology, engineering, and mathematics (STEM) education should be strengthened and that skills relevant to a dynamically changing technology-rich environment should be developed. In this context, societies today and in the foreseeable future are characterized by a new group of skills, which are often called the twenty-first-century skills, or, in other contexts, transversal skills (Greiff et al., 2014). This loosely defined set of skills includes problem-solving, information and communication skills, critical thinking, creativity, entrepreneurship and collaboration. The topic of twenty-first-century skills has become popular in the literature on the future of education (Trilling and Fadel, 2009; National Research Council, 2013; Kong et al., 2014), and a number of projects have been launched to define, assess, and develop these skills.

Although most skills identified under this label are not new in the sense that they have not been studied before or that they have not been relevant in everyday life, the way they are utilized in this century may be novel. The main novelty is that these skills today are mostly used in a technology-rich environment. Therefore, they should be measured by means of technology. This approach is demonstrated by the Assessment and Teaching of twenty-first-Century Skills (ATC21S) project, among other studies. The first phase of the ATC21S project dealt with definitions and psychometric, technological and policy issues (Griffin et al., 2012), while the second phase focused on the assessment of collaborative problem-solving (Griffin and Care, 2015).

Technology-based assessment has a number of advantages over traditional paper-and-pencil tests in a number of respects. Computerized tests, especially assessments delivered online, may make the entire assessment process more reliable and valid, faster, easier, and less expensive. Beyond these general benefits, there are some constructs which could not be measured without computers. There are domains where technology use is central to the definition of the domain (e.g., information-communication literacy and digital reading), while in other cases it would not be possible to implement the assessment process without technology (Csapó et al., 2012). DPS is such a construct, as students interact with computer-simulated systems during the testing process. Technology is the best means not only to assess these skills, but to develop them as well; for example, simulation- and game-based learning may provide an authentic learning environment to practice these skills (see Qian and Clark, 2016).

Those projects whose aim it was to precisely identify the twenty-first-century skills were able to define only a few of them in a measurable format (Binkley et al., 2012). Even fewer of those skills have an established research background that makes it possible to use them in a large-scale project. Of these, problem-solving, both dynamic (Greiff et al., 2014) and collaborative (OECD, 2013b; Griffin and Care, 2015; Neubert et al., 2015), is sufficiently developed for broader practical use. Beyond these strengths, DPS is a good representative of the twenty-first-century skills because, through its component skills, it may overlap with several other complex skills in this group.

### Assessment of dynamic problem-solving

Problem-solving is one of the most commonly noted constructs among the “new” twenty-first-century skills; it also has a long history in cognitive research (see Fischer et al., 2017). By now, cognitive research has identified a number of different types of problem-solving which can be classified by several aspects. Domain-specific problem-solving can be distinguished from the domain-general kind, analytical from complex, and static from interactive. In the present study, we deal with the assessment of dynamic problem-solving, which is interactive and can be considered as a specific form of complex problem-solving. Dynamic problem-solving, as was shown in the previous section, can only be measured by means of technology.

Complex problem-solving has already been studied in a number of contexts; previous research shows that it is a generic cognitive skill, but is different from general intelligence (Funke, 2010; Wüstenberg et al., 2012; Greiff et al., 2013a). Using computers to assess problem-solving has allowed a migration of previous paper-based tests to an electronic platform, thus improving the efficiency and usability of the tests as well as opening up a range of new prospects (Wirth and Klieme, 2003). These new possibilities include constructing more real life-like scenarios, using simulations, offering interactive activities, and in this way improving the ecological validity of the assessments in general.

Using simulation to study problem-solving was already proposed long ago (Funke, 1988), but the broad availability of computers launched a new wave of research based on computer-simulated systems (Funke, 1993, 1998; Greiff et al., 2013a). The difficulty level of tasks based on simulation is easily scalable; even simulated minimal complex systems offer outstanding opportunities to study the processes of problem-solving (Sonnleitner et al., 2012; Funke, 2014; Funke and Greiff, 2017; Greiff and Funke, 2017).

DPS as a specific category of complex, interactive problem-solving offers outstanding potential both to create tests for laboratory studies and for large-scale assessment (Buchner and Funke, 1993; Funke, 2001; Greiff et al., 2012). When students solve dynamic problems on a computer, their activities can be logged and fine mechanisms of their cognition can be explored by analyzing log files (Tóth et al., 2017).

Several types of problem-solving have already been assessed three times within the framework of PISA. First, static problem-solving was assessed in 2003 with paper-based tests (OECD, 2004; Fleischer et al., 2017). Then, in 2012, problem-solving was measured with computerized tests comprising two types of tasks, static (15 items) and interactive (27 items). The static items were similar to those of the PISA 2003 assessment; they were computerized versions of items that would be possible to measure with paper-and-pencil tests as well, while the interactive items were novel in large-scale assessments and measured the same construct (DPS) as the present study, based on the MicroDYN approach, too (Greiff and Funke, 2009; Funke and Greiff, 2017).

The 2012 PISA assessment was the first large-scale assessment of DPS in international context and demonstrated that there were large differences between the participating countries in the problem-solving performance of their students, even if the achievement on the main literacy domains was similar (OECD, 2014). The successful completion of the 2012 PISA DPS assessment has accelerated research in this field and inspired a number of further studies (see Csapó and Funke, 2017a). In PISA 2015, collaborative problem-solving was the innovative domain; collaboration was simulated by human–agent interactions (OECD, 2013b).

Assessments of problem-solving have already proved useful in higher education, but the vast majority of them covered domain-specific problem-solving (e.g., Lopez et al., 2014; Zlatkin-Troitschanskaia et al., 2015). As technology-based assessment instruments become more widely available, such skills have been measured more often, capitalizing on experiences from the computer-based assessment of problem-solving. These assessments may be especially useful when the cognitive outcomes of some innovative instructional methods are measured, such as project methods, problem-based learning and inquiry-based learning.

In the present study, we go further when we explore the possibilities for assessing domain-general problem-solving. Our test is based on the MicroDYN approach (Greiff and Funke, 2009; Funke and Greiff, 2017), which measures the same construct that was measured with several dynamic items in the PISA 2012 assessment (OECD, 2014) and in several other studies (Abele et al., 2012; Molnár et al., 2013, 2017; Frischkorn et al., 2014).

DPS tasks have the same general characteristics. Simulated systems are presented which are based on practical contexts and situations that are easy for the problem-solver to comprehend. The simulated systems show a well-defined behavior, the problem-solver has to manipulate some input variables, and the system responds with changes in output variables. This represents a major difference over paper-based tests, as this sort of a realistic interaction with a responding system cannot be created on paper. The purpose of the interaction is to comprehend the rules that determine the behavior of the system.

In the first phase of completing a DPS test task, students interact with the simulated system, manipulate the values of independent variables, and observe how the changes impact the values of dependent variables. This interactive observation is the *knowledge acquisition phase* (also referred to as the rule identification phase), after which students depict the results of their observations on a concept map. Then, they have to manipulate the variables so that they reach a goal state; this is the knowledge utilization phase (or rule application phase). The results from the two phases are scored separately, and as previous research (e.g., Wüstenberg et al., 2012) has shown, there may be significant differences in performance in the two phases. These dynamic tasks are easily scalable, as the number of input and output variables as well as the relationships between them can be changed.

### Aims and research questions

In the present study, we explore the prospects and value of assessing DPS in higher education. Such a test could later be a useful component of university admissions processes, especially in STEM disciplines, where studies require problem-solving in a technology-rich environment. The context of the study allows for an examination of the relationships between subject matter knowledge and problem-solving.

RQ1: How do matriculation examination results predict problem-solving test performance assessed at the beginning of higher education studies? We assume that the knowledge students possessed at the end of their high school studies (assessed by the matriculation exams) correlates with problem-solving, but the strengths of the relationships with the different domains is still open.
RQ2: What are the relationships between subject matter tests and problem-solving performance measured at the beginning of higher education studies? We assume that although the disciplinary knowledge tests correlate well with problem-solving, problem-solving measures other aspects of knowledge; therefore, it has considerable added value over subject matter tests.
RQ3: Are there differences between students in different disciplines? We assume that those who study within different divisions at the university also differ in their problem-solving skills and in the relationships between problem-solving and other tests.
RQ4:How do students' characteristics and background variables influence their problem-solving performance? We may assume that students' family background, mother's level of education, students' intention to learn and students' learning strategies influence how their problem-solving skills develop.

We expect that the results from these analyses may contribute to improving matriculation examinations as well as to devising better admissions processes.

## Methods

### Participants

Participants in the study were students admitted to a large Hungarian university and starting their studies. The university has 12 divisions (arts, science, medicine, etc.), but they vary in size (number of students). All of the divisions participated in the study, but because of the differences between them, not all analyses are equally relevant for every division.

The population for the study was formed exclusively of students who had just finished their high school studies and immediately applied for admission to the university. They took their matriculation examinations in May, and the assessment for this study was carried out in September of the same year.

The target population was 2,319 students, of whom 1,468 (63.3%) participated in the assessment; 57.7% of them were female. The participation rate by division varied from 28.18 to 74.16%.

Student participation was voluntary; students were notified of their option to take part in the assessment prior to commencing their studies. As an incentive, they received credits for successful completion of the tests.

### Instruments

#### Problem-solving test

Students completed a DPS test based on the MicroDYN approach. Several tests composed of similar tasks based on this model have already been used in other studies in Hungary (Molnár et al., 2013, 2017) but only with younger participants. The test prepared for this study consisted of 20 items with varying difficulty levels.

For example, in the knowledge acquisition phase of an easy item, students had to observe how changing the values of two independent variables (e.g., two different kinds of syrup) impacted the value of one dependent (target) variable (sweetness of the lemonade). They moved sliders on the screen to set the current value for the blue and for the green syrup. The system responded by indicating the resultant sweetness level. Students observed what happened and attempted a new setting, observing the sweetness level with such a setting. They had 180 s for the knowledge acquisition phase in each task. In the knowledge utilization phase, they had to reach the required value of the dependent variable (sweetness) by setting the proper values of the independent variables in no more than 180 s. In a difficult item, students had to comprehend more complex relationships between three independent variables (three different training methods used by basketball players) and three dependent variables (motivation, power of the throw and exhaustion). (For more examples of similar DPS items, see Greiff et al., 2013b; OECD, 2014). The two phases of problem-solving were scored separately. The score for the first (knowledge acquisition) phase was based on how accurately the relationships between the variables were depicted, while the score for the second (knowledge utilization) phase reflected the success with which the dependent variables reached the target state.

The difficulty level of the test was close to the optimal for the whole sample with a 45% mean (SD = 21.74). The reliability (Cronbach's alpha) of the entire test was 0.88. The reliabilities of the two problem-solving phases were also high (knowledge acquisition: 0.84; knowledge utilization: 0.83).

#### Disciplinary knowledge tests

Five disciplinary knowledge tests were prepared for the assessment: Hungarian language and literature (Hungarian, for short, with a strong reading comprehension component), mathematics, history, science, and English as a Foreign Language (EFL). Test content was based on the students' high school studies. The tests covered the major topics of the particular disciplines. Difficulty levels for the tests were adjusted approximately to the intermediate-level standards of the matriculation examination. These tests were prepared by experts practiced in preparing matriculation examination tests. The tests made use of the options made available by computer-based testing; using a variety of stimuli (e.g., texts, images, and animation) and response capture (e.g., entering texts and numbers, clicking, and moving objects on the screen by drag-and-drop). The descriptive statistics for the entire sample and the reliability of the instruments are summarized in Table 1. The reliability coefficients for the tests were good, ranging from 0.88 to 0.96.

**Table 1:** Disciplinary knowledge test: descriptive statistics and reliability coefficients.

**Test**	**Number of items**	**Mean (%)**	**Standard deviation**	**Cronbach's alpha**
Hungarian	126	34.30	9.38	0.90
Mathematics	63	59.79	14.27	0.89
History	161	58.60	11.91	0.93
Science	163	45.31	9.01	0.88
EFL	80	55.70	19.11	0.96

#### Background questionnaire

A background questionnaire was administered to participating students via the same platform as the tests. Data were collected in this way about their matriculation examination results and their learning strategies and SES. To minimize the time devoted to administering the questionnaire, only the most relevant variables were explored, where strong relationships were expected. Family background was represented by mothers' level of education (from primary school to master's degree). Students' commitment to study (intention to learn) was measured with the highest degree they intend to earn (bachelor's, master's or PhD). Two scales for learning strategies that use self-reported Likert scales were adapted from the PISA 2000 assessment (elaboration strategies and memorization strategies, see Artelt et al., 2003).

### Procedures

The assessments were carried out in a large computer room at the university learning and information center. Three 2-h sessions (1 h per test) were offered to the students in the first 2 weeks of the semester. The tests and the questionnaire were administered using the eDia online platform.

Students received detailed feedback on their performance a week after the testing period ended. The feedback contained detailed analyses of their performance in the context of normative comparative data.

Data from the achievement tests were analyzed with IRT models. Plausible values were computed to compare the achievements of the age groups, and Weighted Likelihood Estimates (WLE) were used to compute person parameters. The analyses were performed with the ACER ConQuest program package (Wu et al., 1998). Person parameters were transformed to a 500(100) scale so that the university means were set to 500. MPlus software was used to conduct the structural equation modeling (Muthén and Muthén, 2012).

## Results

In this section, we first answer the research questions by examining the details of the correlations between subject matter knowledge represented in the matriculation examination results and in the test scores from the beginning of studies in higher education. Then, we synthesize the relationships in a path model based on these findings.

### Matriculation examination results as predictors of problem-solving performance

Performance in two phases of problem-solving (knowledge acquisition and knowledge utilization) correlated at the moderate level (*r* = 0.432, *p* < 0.001); therefore, it is worth examining the correlations between the matriculation examination results and the phases of problem-solving separately. Here, we only deal systematically with the three mandatory matriculation examination subjects, as these data are available for all participants, while only a small proportion of students took the exams in a science discipline or EFL as an elective. As few students took the matriculation examinations at the advanced level, this analysis involves the results from the intermediate exams. For a comparison, we have computed the correlations between matriculation examination results and those from the knowledge tests (see Table 2).

**Table 2:** Correlations between the matriculation examination results and those from the tests administered at the beginning of higher education studies.

**Matriculation examination**	**Tests**					
	**Hungarian**	**Mathematics**	**History**	**Knowledge acquisition**	**Knowledge utilization**	**Problem-solving**
Hungarian	0.378^***^	0.071^*^	0.220^***^	n.s.	n.s.	n.s.
Mathematics	0.291^***^	0.656^***^	0.233^***^	0.426^***^	0.273^**^	0.414^***^
History	0.395^***^	0.219^***^	0.503^***^	0.133^***^	n.s.	0.109^**^

* *p < 0.05*,

** *p < 0.01*,

*** *p < 0.001*.

Two major observations stand out from Table 2. First, the mathematics matriculation result (which is based on a paper-and-pencil test with constructed responses) predicts problem-solving much more strongly than those in the two other subjects. Second, knowledge acquisition has a stronger correlation with the matriculation examination results than knowledge utilization does. The mathematics and history matriculation results predict the test results for the same respective subjects well; they are lower for Hungarian, which has no significant correlation with problem-solving. We note that when comparing the correlations, ca.0.05 differences are significant at *p* < 0.05, while ca.0.1 differences are significant at *p* < 0.001 (one-tailed, calculated by the Fisher r-to-z transformation). When we note differences between correlations, they are statistically significant.

### Relationships between subject matter tests and problem-solving

Correlations for the six tests are summarized in Table 3. The correlations between disciplinary knowledge test results are moderate (Hungarian and history with science and EFL) or large, and as expected from the similarities between these subjects, the Hungarian–history and mathematics–science pairs correlate more strongly than other pairs. Mathematics has the strongest correlation with problem-solving, followed by science.

**Table 3:** Correlations for the tests taken at the beginning of higher education studies.

**Subject**	**Hungarian**	**Mathematics**	**History**	**Science**	**EFL**
Mathematics	0.434^***^				
History	0.598^***^	0.409^***^			
Science	0.375^***^	0.529^***^	0.395^***^		
EFL	0.307^***^	0.341^***^	0.337^***^	0.399^***^	
Knowledge acquisition	0.156^**^	0.515^***^	0.228^***^	0.422^***^	0.262^***^
Knowledge utilization	n.s.	0.315^***^	0.095^**^	0.254^***^	0.121^**^
Problem-solving	0.125^**^	0.492^***^	0.192^***^	0.401^***^	0.227^***^

** *p < 0.01*,

*** *p < 0.001*.

These correlations confirm once again that knowledge acquisition is a more decisive component of problem-solving items than knowledge utilization. To examine the details of this relationship, we performed regression analyses with problem-solving and its two phases as dependent variables, using the disciplinary knowledge test results as independent variables (Table 4).

**Table 4:** Regression analyses of problem-solving and its two phases as dependent variables with disciplinary knowledge tests as independent variables.

**Independent variables**	**Problem-solving**			**Knowledge acquisition**			**Knowledge utilization**		
	***R***^**2**^ = **0.286**			***R***^**2**^ = **0.309**			***R***^**2**^ = **0.121**		
	**Beta**	***t***	**Sig**	**Beta**	***t***	**Sig**	**Beta**	***t***	**Sig**
Hungarian	−0.151	−4.896	0.000	−0.141	−4.628	0.000	−0.115	−3.351	0.001
Mathematics	0.420	14.056	0.000	0.423	14.424	0.000	0.284	8.585	0.000
History	0.013	0.429	0.668	0.035	1.145	0.252	−0.013	−0.380	0.704
Science	0.218	7.257	0.000	0.214	7.238	0.000	0.154	4.622	0.000
EFL	0.036	1.329	0.184	0.060	2.265	0.024	0.000	−0.010	0.992

The differences between these analyses confirm previous observations on the role of knowledge acquisition and indicate that it is only mathematics and science whose contribution to the variance explained is significant and positive. Furthermore, even in the cases of knowledge acquisition, ~70% of the variance remained unexplained.

### Differences between students studying within different divisions

As can be expected, there are large differences between the divisions at the university, both in performance on knowledge tests and on problem-solving. Therefore, it is anticipated that problem-solving has different relationships with disciplinary knowledge. To examine these differences, we have chosen two divisions with a large number of students participating in the assessments and with different study profiles. The division that deals with the humanities, known as the Faculty of Arts (Arts, for short), participated with 212 students (65.2% of the population, 71.7% female), and the division that deals mainly with the natural sciences, known as the Faculty of Science and Informatics (Science, for short), was represented with 380 students (64.0% of the population, 32.8% female). They performed differently on each test (Table 5), including problem-solving.

**Table 5:** Differences in achievement among students in the two divisions with different study profiles.

**Division**	**Hungarian**	**Mathematics**	**History**	**Science**	**EFL**	**Problem-solving**
	**Mean**	**Mean**	**Mean**	**Mean**	**Mean**	**Mean**
	**(SD)**	**(SD)**	**(SD)**	**(SD)**	**(SD)**	**(SD)**
Arts	539 (98)	466 (92)	529 (99)	481 (78)	526 (108)	464 (94)
Science	486 (89)	546 (103)	495 (94)	525 (91)	506 (94)	542 (93)
*t*	6.28	−9.59	3.87	−5.87	2.02	−9.38
Sig	*p* < 0.001	*p* < 0.001	*p* < 0.001	*p* < 0.001	*p* < 0.05	*p* < 0.001

Achievement differed according to the expectations for the different study profiles. Students at the Arts Faculty performed better in Hungarian, history and EFL, while Science Faculty students performed better in mathematics, science and problem-solving.

To examine the details of the relations between disciplinary knowledge and problem-solving, we performed the regression analyses separately for the two divisions. Taking into account the decisive role of knowledge acquisition, we present only the results for this phase of problem-solving in Table 6. For comparison, the *R*^2^ were 0.203 (Arts) and 0.217 (Science) for the entire problem-solving test when the same analyses were performed.

**Table 6:** Regression analyses of knowledge acquisition as a dependent variable with disciplinary knowledge tests as independent variables for the two divisions.

**Independent variables**	**Faculty of Arts**			**Faculty of Science**		
	***R***^**2**^ = **0.237 (*****p*** < **0.001)**			***R***^**2**^ = **0.237 (*****p*** < **0.001)**		
	**Beta**	***t***	**Sig**	**Beta**	***t***	**Sig**
Hungarian	0.109	1.343	0.181	−0.143	−2.242	0.026
Mathematics	0.252	3.057	0.003	0.379	6.444	0.000
History	0.065	0.806	0.421	−0.035	−0.577	0.564
Science	0.187	2.340	0.020	0.154	2.789	0.006
EFL	0.006	0.087	0.931	0.165	3.072	0.002

Although the same amount of variance of knowledge acquisition was explained by the same set of independent variables, the contributions of the individual variables are different. Mathematics and science play an important role at both divisions, and the contribution of EFL is also significant at the Faculty of Science.

### Relationships between students' background variables and problem-solving performance

Previous studies (e.g., OECD, 2014) have indicated large difference in problem-solving in a number of dimensions. Here, we explore the differences according to some available background variables.

#### Gender differences

Gender differences are routinely analyzed on large-scale national and international assessments. The PISA studies indicated that Hungarian girls' reading comprehension was significantly better than that of boys, while boys' performance was better in mathematics and there were no significant gender differences in science (OECD, 2016). Female and male students performed differently on problem-solving in this study as well. To provide context to interpret the size of gender difference in problem-solving, the differences on other tests are also indicated in Table 7.

**Table 7:** Gender differences in test performance.

**Test**	**Gender**	**Mean**	**SD**	**Difference**	***t***
Hungarian	M	489	106	−26	−4.54
	F	515	94		
Mathematics	M	534	102	49	8.75
	F	485	94		
History	M	524	105	39	7.03
	F	485	92		
Science	M	518	95	29	5.18
	F	489	101		
EFL	M	513	102	23	3.93
	F	490	97		
Knowledge acquisition	M	597	108	93	14.86
	F	503	111		
Knowledge utilization	M	492	126	62	9.40
	F	430	100		
Problem-solving	M	545	98	78	14.6
	F	467	88		

*All differences are significant at p < 0.01*.

The only test where women outperform men was Hungarian language (in line with the better reading performance of the female students); on all other tests, men performed better. The largest difference was found in favor of men in problem-solving. Here again, knowledge acquisition shows a much larger difference, indicating that this is the more sensitive phase of problem-solving.

#### Mothers' education and intention to learn

The relationship of test performance with students' socio-economic status is a well-known phenomenon, although there are large differences in this respect between countries and also between domains of assessment. International assessment programs (e.g., the PISA studies) usually involve complex indices for this purpose, but we have only one variable to represent students' family background, mothers' education. A further variable that may be interesting in this context is what degree students want to earn (intention to learn). We have found a small (Spearman's rho = 0.182, *p* < 0.001) correlation between these two variables. The correlations of test results with mother's educational level and intention to learn are summarized in Table 8.

**Table 8:** Correlations of performance on the tests with mother's education and intention to learn.

**Test**	**Mother's education**	**Intention to learn**
Hungarian	0.153^***^	0.189^***^
Mathematics	0.145^***^	0.167^***^
History	0.134^***^	0.190^***^
Science	0.161^***^	0.242^***^
EFL	0.192^***^	0.157^***^
Knowledge acquisition	0.084^**^	0.116^***^
Knowledge utilization	n.s.	0.064^**^
Problem-solving	n.s.	0.105^***^

** *p < 0.01*,

*** *p < 0.001*.

There are no large differences between the correlations; all are rather small. Mothers' education has little impact on problem-solving. The correlation of problem-solving with intention to learn is small but still significant; the correlation with knowledge utilization here is also smaller than with knowledge acquisition.

#### Learning strategies

As there are only a few questions in the learning strategies questionnaire, we present the texts and the correlations with the problem-solving achievement for each question. Students' answers to these questions show small but significant correlations with problem-solving (Table 9).

**Table 9:** Correlations of the learning strategies questions with problem-solving performance.

**Question**	**DPS1**	**DPS2**	**DPS**
**ELABORATION STRATEGIES**			
When I study, I try to relate new material to things I have learned in other subjects.	0.129^***^	0.075^**^	0.121^***^
When I study, I figure out how the information might be useful in the real world.	n.s.	n.s.	n.s.
When I study, I try to understand the material better by relating it to things I already know.	0.080^**^	0.104^***^	0.109^***^
When I study, I figure out how the material fits in with what I have learned.	n.s.	0.070^*^	0.069^*^
**MEMORIZATION STRATEGIES**			
When I study, I try to memorize everything that might be covered.	−0.153^***^	−0.091^**^	−0.144^**^
When I study, I memorize as much as possible.	−0.097^**^	n.s.	−0.074^**^
When I study, I memorize all new material so that I can recite it.	−0.183^***^	−0.128^***^	−0.185^**^
When I study, I practice by saying the material to myself over and over.	−0.263^***^	−0.135^***^	−0.236^**^

*DPS1, Knowledge acquisition; DPS2, Knowledge utilization; DPS, dynamic problem-solving*.

* p < 0.05;

** p < 0.01;

*** *p < 0.001*.

The elaboration strategies questions correlate positively with problem-solving, while the memorization strategy questions correlate negatively with it. It is quite clear from the content of the questions that students who prefer conceptual meaningful learning over rote learning are better problem-solvers.

### An integrated model of the relations of knowledge acquisition in dynamic problem-solving

We synthesized the results using structural equation modeling (SEM). Taking into account the observations reported in the previous sections, here we deal only with the knowledge acquisition phase. As the main aim of the present study is to validate the DPS test and to explore its usefulness at the beginning of university studies, we conceived a model by using variables with significant correlations. We assume that students' gender and learning strategies influence their disciplinary test results, while these results (students' actual knowledge) influence achievement in DPS.

**Figure 1:**
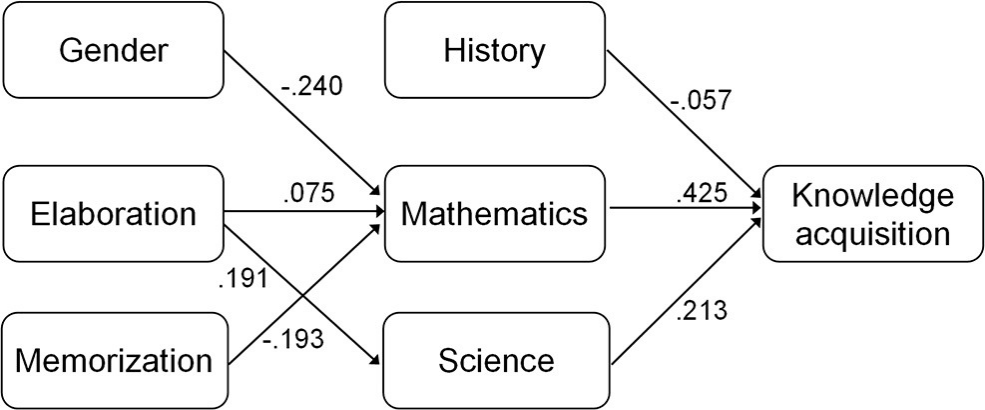
A path model of the relationships with the first phase of problem-solving (knowledge acquisition).

A model that adequately fits the data (RMSEA = 0.046, CFI = 0.986, TLI = 0.949) is presented in Figure 1. Gender influences mathematics test results, while learning strategies have a remarkable impact on mathematics and science. These two disciplines and history have a significant relationship with the first phase of problem-solving.

In this model, positive impacts of elaboration strategies on mathematics and science were found, while success in the knowledge acquisition phase of DPS was positively influenced by science and mathematics. Gender and memorization strategy as well as history have a negative relationship.

## Discussion

Our results confirmed or extended several findings from previous research (e.g., different relationships of the phases of problem-solving) and identified some new relationships as well (e.g., the relationships with learning strategies).

### Determinants of problem-solving achievement at the beginning of higher education studies

Previous research has already identified several characteristics of DPS at different ages, including primary and secondary school students (Molnár et al., 2013), 15-year-old students in the PISA 2012 assessment (which used tests partially built on the MicroDYN approach in a large-scale international comparative survey, OECD, 2014), and university students (Wüstenberg et al., 2012). The present study has shown the feasibility and usefulness of such an assessment in higher education, indicating that DPS is an easily applicable test with several characteristics of the twenty-first-century skills.

Based on the available data, the impact of previous learning was represented by the disciplinary knowledge test results of three matriculation examination subjects. Mathematics had the strongest correlation with problem-solving, which can be explained by the fact that mathematics is studied throughout the 12 years of primary and secondary schooling and by the nature of cognitive processes required by problem-solving (Greiff et al., 2013b; Csapó and Funke, 2017b). The important role of mathematics was also noticed when the correlations with the subject tests were analyzed, and the integrating path model mirrored the same exceptional impact as well.

The first phase of solving dynamic items (knowledge acquisition or rule identification in other studies) has a stronger relationship with any other observed variable than the second phase (knowledge utilization or rule application). Other studies have found similar differences, although the dominance of knowledge acquisition was not so obvious (Wüstenberg et al., 2012). The important role of the first phase, indicated by larger correlations, may be attributed to the kind of reasoning this phase requires. Students have to combine the different values of the independent variables they manipulate in this phase (combinatorial reasoning), judge certain probabilities (probabilistic reasoning) and abstract rules from the observed behavior of the simulated system (inductive reasoning). This may also explain the strong connection (especially of the first phase) to mathematics, as this kind of reasoning is mostly applied when learning mathematics. Rule induction connects DPS to general intelligence as well, as most intelligence tests use inductive reasoning items. Nevertheless, previous research has indicated that problem-solving explains added variance of students' school achievement (GPA) beyond intelligence tests (Wüstenberg et al., 2012), and moderate to large correlation (*r* = 0.44, 0.52, and 0.47 in Grades 5, 7, and 11) has been found between problem-solving and inductive reasoning (Molnár et al., 2013).

Our analyses showed that there were differences between the students preparing for studies in different disciplines both on the level of problem-solving achievement and in the strengths of correlations with domain-specific knowledge. However, some main tendencies, e.g., the dominant role of mathematics and science and the role of the knowledge acquisition phase, may be generalized.

Large gender differences were found on all the tests we used in this study, but the largest one was observed in problem-solving (78 points), mathematics being the second largest one with a much lower difference (49 points). The difference in knowledge acquisition is especially high (93 points). In PISA 2012, gender differences in problem-solving varied from country to country. The OECD mean was 7 points, and in Hungary it was below average, though it was not significant, only 3 points (OECD, 2014). To interpret this discrepancy between the PISA results and the present study, it is worth noting that the Hungarian PISA problem-solving results were below average (459 points) and that not all items were dynamic. Furthermore, in our study, women are overrepresented in the Arts Faculty and in humanities studies in general, while they are underrepresented in STEM studies.

Although there are large differences between students according to the socio-economic status (SES) of their family—and Hungary belongs to a group of countries where the impact of SES is especially strong—there was no large effect found in problem-solving in the PISA 2012 survey. In our study, we also found a modest impact of mothers' level of education on students' problem-solving performance. The fact that problem-solving is less determined by social background than domain-specific competencies indicates a potential opportunity for disadvantaged students as they may show their strengths on these kinds of assessments.

Previous studies have indicated a strong relationship between low- and high-achieving high school students and the different learning strategies they use (Yip, 2013). Our results confirm this notion, as there are clear links between learning strategies and knowledge acquisition in problem-solving. A positive effect of elaboration strategies may have been predictable, but a measurable negative impact of memorization strategies is somewhat unexpected. These results suggest the conclusion that problem-solving is learnable and point to one of the directions in the search for proper training methods. In general, there are two main directions for facilitating the development of this kind of general cognitive skill in a school context. The first is a holistic approach, when developmental impacts are embedded in other educational activities, in this case in learning science and mathematics through meaningful elaborative strategies. Discovery learning and inquiry-based teaching methods may have an impact on the development of problem-solving as well. The second method improves problem-solving by developing component skills (Csapó and Funke, 2017b). We have identified potential component skills; providing training in them may also influence the development of problem-solving.

The results of the SEM indicate the complex nature of the relationships between the variables being explored. The DPS tasks are constructed so that completing them requires no preliminary knowledge within any discipline. Therefore, we may assume that if there are relationships between disciplinary knowledge tests and DPS tests, these relationships are established by factors other than the factual knowledge represented in the knowledge tests. Such factors may be learning strategies (we have variables for representing them) and certain cognitive skills needed both for completing the disciplinary tests and the DPS tests (in this study, we have no variables to represent them in the SEM). In this model, gender as a variable (most probably) mediates women's better reading and poorer mathematics achievement (shown by other studies, e.g., PISA). In sum, this model indicates that men outperform women, and this impact is mediated by the higher mathematics performance among men. The negative impact of memorization is transmitted via mathematics and science.

### Limitations of the present study

As the PISA 2012 assessments also indicated, there are large differences between countries not only on the level of problem-solving performance, but also in the strengths of the relationships between several relevant variables as well; therefore, some particular results found in one country cannot be generalized over countries and cultures. Although some general tendencies were found, we have seen that the strength of the relationships we have examined in this study differs by division. Therefore, the generalizability of the strengths of these relationships is limited; nevertheless, the method we applied in this study is generalizable and may be useful to explore the actual relationships in any higher educational context. Participation was voluntary in the study; the actual samples are thus not representative of the divisions. Nevertheless, the analyses revealed some generalizable tendencies as well.

### Conclusions: further research and prospects for assessment supporting high school–university transition

The results from the present study have raised several further questions worth researching. The dominant role of knowledge acquisition indicates a promising line of inquiry to explore this phase in more detail. One promising direction is to identify students' knowledge acquisition strategies, e.g., the way they manipulate the independent variables when they attempt to discover how these manipulations impact changes in the dependent variable. Students' activities are logged, and their strategies may be ascertained with log file analyses. Latent class analysis may be an effective method to identify students' exploring strategies.

The knowledge acquisition phase also deserves further study from the perspective of its relationships to learning strategies as well, for example, examining if poor problem-solving performance can be an indicator of inadequate learning strategies. If such a connection can be proven, problem-solving assessment could be a diagnostic tool for identifying poor leaning strategies, possibly more reliable than self-reported questionnaires. Further insights into the nature of cognition in the knowledge acquisition phase could be expected from studying it in relation to the learning to learn assessments (Greiff et al., 2013b; Vainikainen et al., 2015).

Several skills may be identified which are needed to successfully complete phases of problem-solving. A systematic examination of the role of some supposed component skills (e.g., combinatorial reasoning, probabilistic reasoning, correlational reasoning and inductive reasoning) would provide foundations for the development of problem-solving by strengthening its component skills as well.

The results from this study indicated that technology-based assessment of problem-solving may be a useful instrument to moderate the secondary–tertiary education transition. To improve its usefulness, the scoring system may be further developed, extending it with an automated log file analysis. Such an instrument would be especially helpful in selection processes (admissions tests) for the STEM disciplines. More detailed analyses of the relationship between problem-solving and the study profile would be needed to improve the test. In the present study, we compared divisions of study within the university, but a division is still not homogeneous; for example, students in biology training may be different from those in mathematics.

We have found significant positive relationships with the questions on elaboration learning strategies and negative relationships with the questions on memorization strategies. In the present study, there were not enough questions to use sophisticated scales for representing these learning strategies, but the findings indicate the relevance of exploring the role learning strategies play in the development of problem-solving. This seems a promising area both for research and practice not only for higher education but also for earlier phases of school education.

The predictive power of DPS can be explored later when data is available on the university achievement of the students participating in the present assessment. The test may have a diagnostic value (indicating poor study strategies or insufficient problem-solving skills) and can also be used to aid students in selecting a study track better suited to their cognitive skills.

## Author contributions

BC and GM have made an equal contribution to the study, including the design, data collection, analyses, and writing of the manuscript, and have both approved it for publication.

### Conflict of interest statement

The authors declare that the research was conducted in the absence of any commercial or financial relationships that could be construed as a potential conflict of interest. The reviewer, CL, and handling Editor declared their shared affiliation.
